# Mechanistic patterns and clinical implications of oncogenic tyrosine kinase fusions in human cancers

**DOI:** 10.21203/rs.3.rs-3782958/v1

**Published:** 2024-01-17

**Authors:** Roberto Chiarle, Taek-Chin Cheong, Ahram Jang, Qi Wang, Giulia Leonardi, Biagio Ricciuti, Joao Alessi, Alessandro Di Federico, Mark Awad, Maria Lehtinen, Marian Harris

**Affiliations:** Boston Children’s Hospital and Harvard Medical School; Harvard Medical School; Boston Children’s Hospital and Harvard Medical School; Boston Children’s Hospital and Harvard Medical School; Boston Children Hospital; Dana-Farber Cancer Institute; Dana-Farber Cancer Institute; Dana-Farber Cancer Institute, Harvard Medical School; Dana Farber Cancer Institute; Boston Children’s Hospital; Boston Children’s Hospital and Harvard Medical School

## Abstract

Tyrosine kinase (TK) fusions are frequently found in cancers, either as initiating events or as a mechanism of resistance to targeted therapy. Partner genes and exons in most TK fusions are typical and recurrent, but the underlying mechanisms and clinical implications of these patterns are poorly understood. Here, we investigated structures of > 8,000 kinase fusions and explore their generative mechanisms by applying newly developed experimental framework integrating high-throughput genome-wide gene fusion sequencing and clonal selection called Functionally Active Chromosomal Translocation Sequencing (FACTS). We discovered that typical oncogenic TK fusions recurrently seen in patients are selected from large pools of chromosomal rearrangements spontaneously occurring in cells based on two major determinants: active transcription of the fusion partner genes and protein stability. In contrast, atypical TK fusions that are rarely seen in patients showed reduced protein stability, decreased downstream oncogenic signaling, and were less responsive to inhibition. Consistently, patients with atypical TK fusions were associated with a reduced response to TKI therapies, as well as a shorter progression-free survival (PFS) and overall survival (OS) compared to patients with typical TK fusions. These findings highlight the principles of oncogenic TK fusion formation and their selection in cancers, with clinical implications for guiding targeted therapy.

Tyrosine kinase (TK) gene fusions are common genetic alterations across the cancer types, including both hematologic and solid cancers. They are one of the earliest genomic events that initiate oncogenesis, as demonstrated in functional models^1^, as well as in cancer genome studies^2–4^. Furthermore, acquisition of TK fusions, such as *ALK* or *RET* fusions, have been also reported during targeted therapy in non-small cell lung cancer (NSCLC) and other tumors, as a mechanism of resistance^5–9^. Identification of the functional TK fusions is crucial in the clinic because small-molecular TK inhibitors are highly effective for patients with cancers harboring these TK fusions, often regardless of the tissue of origin^10–12^.

TK genes typically fuse with a partner gene that provides an active promoter for the fusion gene’s expression and dimerization or oligomerization domains for TK activation through the fused TK domain^13^. Mechanistically, TK fusions are often formed by genomic rearrangements between two DNA double-strand breaks (DSBs) in introns, leading to the transcription of in-frame chimeric gene products. However, the mechanisms by which these recurrent breakpoints are selected among the large pool of potential fusion combinations remain unclear^14^. For example, in patients with NSCLC, *EML4* is the most frequent partner gene of *ALK* fusions and *CD74* for *ROS1* fusions. However, *EML4-ROS1* or *CD74-ALK* fusions have not been reported, although these fusions can theoretically be functional. Furthermore, multiple introns in *ALK* can potentially create in-frame *ALK* fusions fully preserving the kinase domain, but most *ALK* fusions involve breaks in intron 19, regardless of its partner genes^15,16^. The molecular basis of selecting partner genes, introns, and the clinical implications of different fusion types between the typical and atypical fusions remain unclear.

In this study, we identify mutually exclusive fusion partner selection and specific exon usage in TK fusions based on the Catalogue of Somatic Mutations in Cancer (COSMIC) datasets. We develop a new experimental framework integrating high-throughput genome-wide gene fusion sequencing followed by clonal selection under the pharmaceutical selective pressure, which we call Functionally Active Chromosomal Translocation Sequencing (FACTS). Through this approach, we identify oncogenic TK fusions that spontaneously occur in the NSCLC cells and confer selective advantages. Furthermore, we determine the critical role of gene transcription and protein stability to explain the recurrent selection of the typical TK fusions. Finally, we highlight their clinical implications that impact the outcome of patients during TKI treatment.

## Results

### Characterization of kinase fusions across cancer types

We analyzed 8,805 3’ kinase gene fusions curated from the COSMIC, focusing on the seven most common kinase fusions involving *ALK, RET, ROS1, NTRK1, NTRK3, ABL1*, and *BRAF* genes found in various types of cancers (**Supplementary Table 1**). In this study, we used 7,751 kinase fusions where the mRNA junction positions were validated (**Supplementary Table 1**).

**Figure 1:**
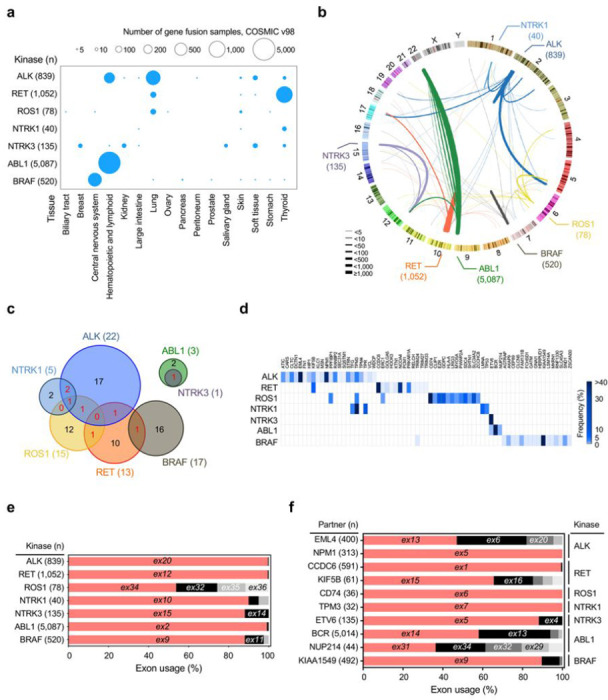
Landscape and characterization of recurrent 3’ kinase fusions across multiple cancers curated from COSMIC v98. **a**, The dot plot indicates the distribution of kinase fusion in each tissue type. The size of the dot corresponds to the number of kinase fusions in each tissue type. **b**, Circos plot showing the genome-wide distribution of 3’ kinase fusions. The thickness of arcs represents the number of fusions of each kinase fusions and each color corresponds to fusions with each kinase gene. **c**, Venn diagrams showing the overlap of fusion partners shared between *ALK* (n=22 partner genes), *NTRK1* (n=5), *ROS1* (n=15), *RET* (n=13), *BRAF* (n=17), *ABL1* (n=3), and *NTRK3* (n=1) fusions. **d,** Frequency of kinase fusion partners. **e and f,** Exon usages of kinase genes (**e**) and partner genes (**f**) in each 3’ kinase fusions.

We first analyzed the prevalence of the seven 3’ kinase fusions across 16 tissue types. Except for ABL1 fusions, all kinase fusions were identified in multiple tissue types at variable frequencies between the tissue types (Fig. 1a **and Supplementary Table 2**). We observed several common patterns of kinase fusions, in terms of partner genes and intron usage. ALK fusions were predominantly observed in lung cancer and lymphoma, while RET, ABL1, and BRAF fusions have been identified most frequently in thyroid cancer, leukemias, and pediatric low-grade gliomas, respectively. *EML4* was the most frequent partner of *ALK* fusions in lung cancers, while *NPM* was the case in lymphomas (Fig. 1b **and Supplementary Table 1**). The other TK fusions, including *RET, ROS1, BRAF*, and *ABL1* fusions, showed several frequent partner genes (Fig. 1b **and Supplementary Table 3**). Partner genes were largely specific to kinase genes, with several exceptions (Fig. 1c). For example, seven partner genes (9.6%), including *KIF5B, TPM3, ERC1*, and *ETV6*, were shared across the 73 kinase fusions (Fig. 1c, d **and Supplementary Table 1**). In contrast, most frequent partner genes, i.e., *EML4, CCDC6, CD74, BCR* and, *KIAA1549* were exclusively associated with *ALK, RET, ROS1, ABL1* and *BRAF* fusions, respectively (Fig. 1c, d **and Supplementary Table 3**). In summary, this analysis shows tissue type- and kinase gene-specific partnering in fusion oncogene formation.

Next, we analyzed the locations of fusion events between the kinase and partner genes. Notably, most fusion events in *ALK, RET*, and *ABL1* occurred at the 5’ end of exon 20 (e20; 99.6%, 837/839), e12 (99.5%, 1,047/1,052), and e2 (99.1%, 5,040/5,087), respectively, regardless of their fusion partners. In contrast, *ROS1* fusions occurred at variable locations, including e32 (20.5%), e34 (53.8%), e35 (14.1%), and e36 (11.5%) (Fig. 1e **and Supplementary Table 3**). A similar pattern was observed in the partner genes. Several partner genes showed exclusive preference in exon usage. For example, all *NPM1-ALK* and *CD74-ROS1* fusions used a specific exon in the partner genes (e5 of NPM1 and e6 of CD74). In contrast, *EML4* and *KIF5B* used several exons, including e6, e13, and e20 for *EML4* and e15 and e16 for *KIF5B* (Fig. 1f **and Supplementary Table 3**). This indicates potential selective benefit of specific exon usage in creating kinase fusions.

### Functionally Active Chromosomal Translocation Sequencing (FACTS) identifies oncogenic ALK fusions genome-wide

Genome-wide techniques identifying chromosomal translocations, such as HTGTS and TC-seq, have been widely used to study the mechanisms of translocation in normal and tumor cells by cloning chromosomal junctions generated within a few days of inducing a programmed DNA DSB at a specific site^17–19^. However, not all these translocations result in functional fusion genes, and their impact on oncogenesis cannot be determined by these technologies alone. To overcome this limitation, we developed FACTS to specifically map functional oncogenic fusions genome-widely in the setting of pharmaceutical selective pressure. Inspired by the recent reports indicating fusion oncogene formation as a mechanism of resistance to EGFR inhibitors, we applied FACTS into the *in vitro* model of EGFR inhibitor resistance using PC-9 cells, harboring *EGFR*-activating mutation (*EGFR* E746-A750del)^20^.

PC-9 cells have been extensively used to characterize various mechanisms of resistance to EGFR inhibitors. Under the EGFR inhibitor treatment, PC-9 cells typically undergo cell-cycle arrest, and a small number of cells undergo persistence. Emergence of fully resistant clones require additional genetic alterations, including secondary mutations in EGFR that prevent TKI binding^21^ or another oncogenic driver events that bypasses EGFR inhibition, such as MET amplifications or the acquisition of fusion oncogenes^22^. Therefore, we reasoned that PC-9 cells under selective pressure from a selective EGFR inhibitor osimertinib^23^ would be an ideal setting to test the functional outcome of various kinase fusions induced by genome-wide translocations.

**Figure 2:**
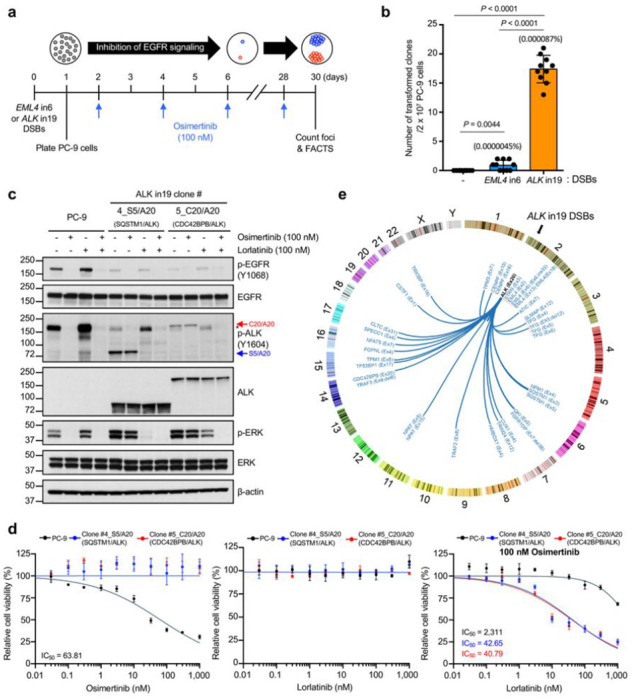
Spontaneously formed ALK fusions induce resistance to osimertinib in PC-9 cells. **a**, Experimental timeline to select osimertinib-resistant clones in PC-9 cells. **b**, Quantification of osimertinib-resistant clones in PC-9 cells treated as in (**a**). Data show means of ten biological replicates, with error bars representing ±s.e.m; significance was determined by an unpaired, two-tailed Student’s *t*-test. **c**, Representative western blots of the signaling changes in osimertinib-resistant clones treated with EGFR inhibitor (osimertinib) and/or ALK inhibitor (lorlatinib). Red and blue arrows represent phosphorylated CDC42BPB-ALK and SQSTM1-ALK fusion proteins, respectively. Asterisk represents a non-specific band. Similar results were observed in two independent experiments. **d**, Sensitivity to osimertinib (**left**), lorlatinib (**middle**), and combination of osimertinib plus lorlatinib (**right**) in osimertinib-resistant clones. Data show means of three biological replicates, with error bars representing ±s.e.m. **e**, Circos plot showing the genome-wide distribution of ALK fusion partners identified in osimertinib-resistant PC-9 cells. Arcs represent functional rearrangements joining *ALK* (exon 20) to the indicated fusion partner.

ALK fusions are the most frequent TK fusions found in 3–7% of patients with NSCLC^15^ and drive resistance to targeted therapy in patients with EGFR-mutant or KRAS^G12C^-mutant cancer^7–9^. Consistently, PC-9 cells expressing two sgRNAs targeting the relevant introns in *EML4* (intron 6 or intron 13) and *ALK* (intron 19) to force the formation of typical EML4-ALK fusions generated resistant clones expressing the EML4-ALK fusions at a frequency of 1.16%-1.34% upon osimertinib selection (**Extended Data** Fig. 1a-d), which was consistent with the frequency of translocations induced by two DNA DSBs in previous studies^24,25^. Furthermore, *EML4-ALK* fusion expressed from the endogenous *EML4* promoter rapidly induced osimertinib resistance in PC-9 cells by producing active and phosphorylated EML4-ALK fusion proteins (**Extended Data** Fig. 1e). Next, we applied FACTS to study ALK fusion formation from a single programmed DSB with any partners in the genome under the osimertinib treatment. We induced a programmed DSB in intron 19 of *ALK*, the hotspot of genomic rearrangements causing EML4-ALK fusions in NSCLC^15^ (Fig. 2a). Multiple osimertinib-resistant clones developed during selection, with an estimated frequency of ~ 1 clone/million cells (Fig. 2b). Analysis of single clones showed that each expressed ALK proteins with a wide range of sizes from approximately 60 kDa to 400 kDa, likely indicating formation of fusion oncoprotein in different sizes (**Extended Data** Fig. 1f). In contrast, when we introduced a DSB in intron 6 of *EML4* gene, no ALK expression was observed in osimertinib-resistant clones (**Extended Data** Fig. 1g), indicating that the DSB at *ALK* is critical in fusion formation. The ALK fusions formed after the DSBs at ALK intron 19 showed a strong phosphorylation of the kinase domain, which resulted in sustained activation of the MAPK pathway despite the presence of osimertinib (Fig. 2c), explaining the mechanism of resistance. Both ALK and ERK1/2 phosphorylation were completely blocked by adding ALK-specific inhibitor lorlatinib (Fig. 2c). Consistently, the growth of osimertinib-resistant clones was inhibited by lorlatinib (Fig. 2d). These data showed that a DSB in *ALK* led to the formation of in-frame fusions, of which expression conferred resistance to osimertinib.

Next, we identified unknown 5’ partner genes of these ALK fusions by using a 3’ end-directed fusion assay^26^. Several in-frame ALK fusions were identified (Fig. 2e **and Supplementary Table 4**), and a subset of them was further validated in single clones using RT-PCR (**Extended Data** Fig. 1h). Three of these fusion partners, *EML4, STRN*, and *ATIC*, are on chromosome 2, where *ALK* is located, whereas others were spread in the genome (Fig. 2e **and Extended Data** Fig. 1i). Remarkably, several of these spontaneous ALK fusions were identical to those described in NSCLC^27^ or in other tumor types^4^ (**Supplementary Table 4**). For example, *EML4-ALK* fusions joined e2, e6, e13, or e18 of the *EML4* gene to e20 of the *ALK* gene (Fig. 2e **and Supplementary Table 4**), exactly as seen in patients with NSCLC and other tumors^28,29^. In addition, some fusion partners showed exclusive exon usage, such as e3 in *STRN* and e31 in *CLTC*, while others showed variable usage (Fig. 2e **and Supplementary Table 4**). These exon usages were identical to the corresponding ALK fusions found in NSCLC and thyroid cancer (**Supplementary Table 4**). We also identified a list of new ALK fusions that have not been described in human tumors (**Supplementary Table 4**), which may indicate rare functional fusion events yet to be discovered. Several of them (e.g., *QKI, TRAF2, TRAF3*, and *TP53BP1*) were the genes previously reported in fusions with other kinases^30–32^ and contain dimerization or oligomerization domains, further supporting their functionality (**Extended Data** Fig. 1j **and Supplementary Table 4**). Taken together, FACTS demonstrated functional fusion oncogene formation through genome-wide translocations after a single DSB at *ALK* intron 19 and reproduced *ALK* fusion landscape in human cancers.

To test whether oncogenic ALK fusions can also be generated in non-cancerous cells, we applied FACTS to the bronchial epithelial BEAS-2B cells. These cells can grow *in vivo* once they are transformed by oncogenic drivers^33^. As a positive control, we injected mice with BEAS-2B cells where two DSBs were induced in *EML4* intron 6 or 13 and *ALK* intron 19. As expected, all mice in this group developed tumors (**Extended Data** Fig. 1k-m). When we injected mice with BEAS-2B where a single DSB was introduced in *ALK* intron 19, we observed tumor formation at a lower rate and with a slower growth kinetics (**Extended Data** Fig. 1l, m). FACTS and RT-PCR validation revealed that tumors expressed various ALK fusions identical to those in PC-9 cells and patient samples (**Extended Data** Fig. 1n **and Supplementary Table 4**). Protein expression of these fusions was most likely determined by the fusion partner, with some fusions being expressed at higher levels than others (**Extended Data** Fig. 1o, p). Thus, by applying FACTS to immortalized normal-like bronchial epithelial cells, we demonstrated that a single DSB in *ALK* intron 19 produced functional ALK fusion oncogenes that led to a malignant transformation *in vivo*.

### FACTS identifies oncogenic RET, ROS1, and NTRK1 fusions

We next applied FACTS to other kinase fusions. *RET, ROS1*, and *NTRK* family gene fusions are found in approximately 4% of patients with NSCLC^34^. We designed FACTS by introducing one DSB in their intron that is most frequently involved in chromosomal translocations, (i.e., intron 11 for *RET*, intron 33 for *ROS1*, and intron 11 for *NTRK1*; **Extended Data** Fig. 2a-d). Resistant clones developed after 4 weeks of osimertinib selection at a frequency comparable to what was observed from clones with ALK fusion (Fig. 2b **and Extended Data** Fig. 2e). FACTS identified several in-frame chimeric proteins with RET, ROS1, and NTRK1 and their joined partners across the genome (**Extended Data** Fig. 2f-n **and Supplementary Table 4**). We validated some of these acquired fusions by RT-PCR and Sanger sequencing and confirmed that they were identical to the *RET* fusions described in patients with NSCLC (**Extended Data** Fig. 2o, p). Other fusions were novel and not yet described in patients (**Supplementary Table 4**). We confirmed that acquired RET fusions conferred resistance to osimertinib, as demonstrated by the reversal of resistance phenotype by selpercatinib (**Extended Data** Fig. 2q). The fusion partner genes identified here also contained dimerization or oligomerization domains (**Extended Data** Fig. 2i-k **and Supplementary Table 4**). Intriguingly, FACTS identified exon fusion variants involving different *ROS1* exons (e34, e35, and e36) or *NTRK1* exons (e12 and e13) but only e12 of *RET* (**Extended Data** Fig. 2f-h **and Supplementary Table 4**), which is consistent with what was reported from patients^27,35–37^.

### Gene transcription, rather than chromatin accessibility, dictates the selection of partner genes in TK fusions

Next, we investigated how ALK fusions are selected among many potential rearrangements, and which mechanistic factors dictate the choice of ALK fusion partners in the genome. Among all reported partner genes of ALK fusion from the patients with NSCLC, those identified by FACTS in our PC-9 model showed a significantly higher level of transcription (**Extended Data** Fig. 3a, b). In contrast, we found no difference in terms of chromatin accessibility measured by ATAC-seq or histone activation marks between the FACTS-identified and -unidentified genes (**Extended Data** Fig. 3c, d). Recurrent translocation partners consistently showed active chromatin marks (**Extended Data** Fig. 3e-j). These findings were consistent in partner genes of RET, ROS1, and NTRK1 fusions^35–37^ (**Extended Data** Fig. 3k-p). Taken together, the partner genes selected in FACTS were associated with higher level of transcription, compared to the other partner genes not identified by FACTS but reported in patients.

Next, we further explored whether gene transcription was sufficient to induce the formation of oncogenic fusions. PC-9 cells express very low to undetectable levels of HLA-DR molecules and the invariant chain CD74 that is essential for the assembly and subcellular trafficking of the MHC class II complex^38^ (**Extended Data** Fig. 4a-d). We hypothesized that this undetectable expression could explain why CD74 or HLA-DR fusions with kinases^39^ were not identified by FACTS in PC-9 cells. Because expression of both CD74 and HLA-DR can be induced by the Class II transactivator (CIITA)^40^ (**Extended Data** Fig. 4e), we asked whether induction of *HLA-DR* or *CD74* expression by CIITA was sufficient to generate fusions of HLA-DR or CD74 with ROS1. FACTS was applied to PC-9 cells expressing CIITA that showed significantly increased HLA-DR and CD74 mRNA and protein levels (**Extended Data** Fig. 4f-m). By introducing DSBs in intron 33 of *ROS1* (**Extended Data** Fig. 5a), we identified genome-wide oncogenic fusions including HLA-DRB1-ROS1 fusions in which the breakpoint in the *HLA-DRB1* gene was identical to that observed in patients with HLA-DRB1-MET fusion^39^ (**Extended Data** Fig. 5b, c **and Supplementary Table 5**). We estimated the frequency of HLA-DRB1-ROS1 fusions at 6.7% using single clone analysis (**Extended Data** Fig. 5d). In contrast to HLA-DRB1-ROS1 fusions, CD74-ROS1 fusions were not detected, which suggests that induction of transcription for the *CD74* gene was not sufficient to trigger CD74-ROS1 translocations. However, when we simultaneously introduced DSBs in both *CD74* and *ROS1* genes in either control PC-9 or CIITA-expressing PC-9 cells, resistant clones rapidly emerged only in CIITA-expressing PC-9 cells (**Extended Data** Fig. 5e, f). While DNA junctions were detected in both cells, CD74-ROS1 fusion transcripts were detected only in CIITA-expressing PC-9 cells (**Extended Data** Fig. 5g-i). These results suggest that increased gene expression of the partner gene is sufficient to induce the formation of TK fusions in loci such as HLA-DRB1, which is located on the same chromosome with ROS1, and point out that the detection of DNA junctions is insufficient to determine oncogenicity of resulting TK fusions without evidence of efficient transcription of the TK fusion.

### Oncogenic TK fusions originate after selection of pools of rearrangements spontaneously occurring in fusion partner and TK genes

**Figure 3:**
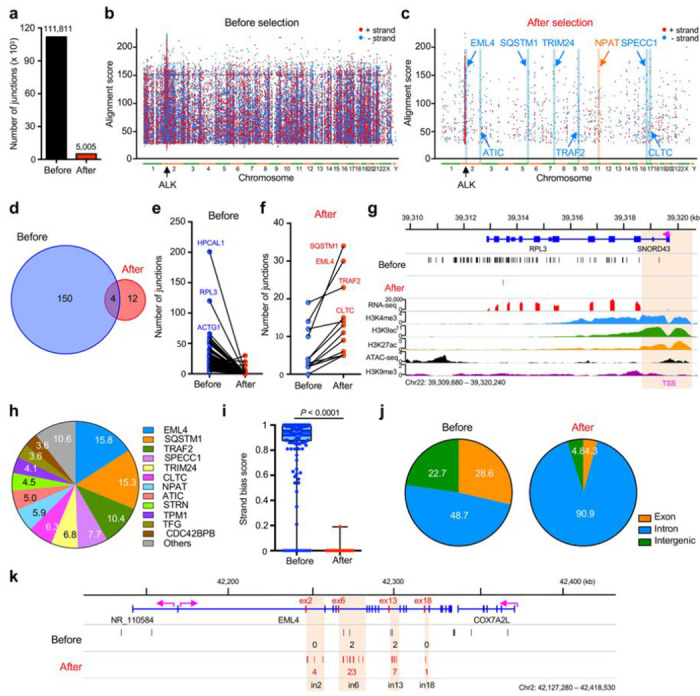
Characterization of functional ALK fusions identified in osimertinib-resistant PC-9 cells by HTGTS. **a**, The total number of translocations in before and after osimertinib selection by HTGTS. **b and c**, Rainfall plots showing genome-wide distribution of translocations before (**b**) and after (**c**) osimertinib selection in PC-9 cells. Each dot represents a single DNA translocation ordered on the X-axis according to its position in the human genome. Red and blue dots represent the orientation of DNA translocations on chromosome plus or minus strand, respectively. Data pooled from six biological replicates. **d**, Venn diagram showing the overlap of shared DNA translocation hotspots between before and after selection. CRISPR off-targets were excluded. **e and f**, Change in the number of DNA translocations in hotspots before (**e**) and after (**f**) osimertinib selection. **g**, Distribution of DNA breakpoints and histone modification marks in *RPL3* gene. Transcription start site (TSS) is indicated in pink. **h**, Frequency of ALK translocations with the indicated partner genes. **i**, Box plot showing the strand bias score of DNA translocations before and after osimertinib selection. Y-axis represents strand bias score where 1 indicates no strand bias and 0 a 100% strand biased. **j**, Pie graphs showing translocation distributions analyzed from hotspots identified before (**left**) and after (**right**) osimertinib selection. **k**, Detailed distribution of DNA breakpoints in *EML4* genes. The purple arrows indicate orientation of genes. The number of translocations in focal clusters is indicated in black and red for before and after selection, respectively.

In some tumors such as lymphoma, recurrent translocations are the result of the activity of the activation-induced cytidine deaminase (AID) enzyme that targets specific regions of the genome^41^. Therefore, we asked whether the selection of specific partners or exons by TK fusions is mechanistically determined by the formation of DSBs in specific positions of genes or rather by the selection of random genomic DSBs. To this end, we generated libraries of DNA junctions by HTGTS which allows for an unbiased detection of genome-wide chromosomal rearrangements before selection^17^ (**Extended Data** Fig. 6a, b). HTGTS yielded 111,811 genomic translocation breakpoints before selection, which were distributed throughout the genome with enhanced clustering in the 2 Mbp regions surrounding the *ALK* DSB (Fig. 3a-c **and Extended Data** Fig. 6c). We identified 154 hotspots with significantly enriched breakpoint clustering (Fig. 3d **and Supplementary Table 6**). Only 2.6% (4/154; *EML4, SQSTM1, TRAF2*, and *CLTC*) of these hotspots occurred in genes that are known partners of ALK fusions (Fig. 3d **and Supplementary Table 6**). In sharp contrast, HTGTS performed with resistant clones after osimertinib selection yielded 5,005 DNA breakpoints with 81% hotspots (13/16) occurring in genes leading to the transcribed ALK fusions identified by FACTS (Fig. 2e and Fig. 3a **and Supplementary Tables 4 and 6**). Several strong genomic translocation hotspots observed before selections completely disappeared after selection (Fig. 3e-g and **Extended Data** Fig. 6d), most likely because the resulting rearrangements did not generate a functional *ALK* fusions. *EML4* was the gene most frequently translocated with *ALK* after selection (Fig. 3h). Breakpoints before selection did not show a preferential strand bias, which is consistent with previous works of genome-wide cloning of unselected translocations^17,42^. In contrast, the breakpoints after selection showed a strong bias for an orientation of the gene leading to a functional fusion with *ALK* (Fig. 3i), with DNA breakpoints markedly enriched for junctions occurring in gene introns (Fig. 3j). Within individual partner genes, we observed a selective enrichment of breakpoints occurring in introns leading to in-frame functional fusions with ALK (Fig. 3k **and Extended Data** Fig. 6e-h). Overall, these data indicate that the formation of ALK fusion is the result of a functional selection of transcribed translocations based on the location and orientation, not just a reflection of DNA break frequency.

Next, we focused on TK genes and generated HTGTS libraries in BEAS-2B and PC-9 cells by inducing a DSB in *EML4* as bait to capture breaks spontaneously occurring in TK genes (**Extended Data Fig. 7a**). We looked at the distribution of breakpoints in *ALK, RET, ROS1, NTRK1*, as well as other kinase genes known to generate oncogenic fusions in lung cancer, such as *EGFR, ERBB4, MET, FGFR3*, and *EPHA2*^43^. Breakpoints identified in these kinases were evenly spread throughout the gene body including introns and exons without clear clusters (**Extended Data Fig. 7b-j**). In both BEAS-2B and PC-9 cells, more breakpoints were observed in *ALK* than in other kinases (**Extended Data Fig. 7k, l and Supplementary Table 7**), most likely because *EML4* and *ALK* are proximally located on the same chromosome 2^44,45^. More breakpoints in *EGFR* were detected in PC-9 cells than in BEAS-2B cells (**Extended Data Fig. 7f, k, l**), most likely due to the presence of > 4 copies of the *EGFR* gene in PC-9^22^, and frequent breakpoints were observed also in *EPHA2* gene, which is highly transcribed in these cells (**Extended Data Fig. 7j, l**). All combined, these data suggest that the preferential usage of specific partners or exons during oncogenic TK fusion formation is the result of a selection process among multiple combinations of junctions created by DSBs spontaneously generated in the genome, rather than due to the presence of pre-existing clusters of breakpoints like in the case of AID-initiated translocations.

### Protein stability determines the selection of TK fusion partners

**Figure 4:**
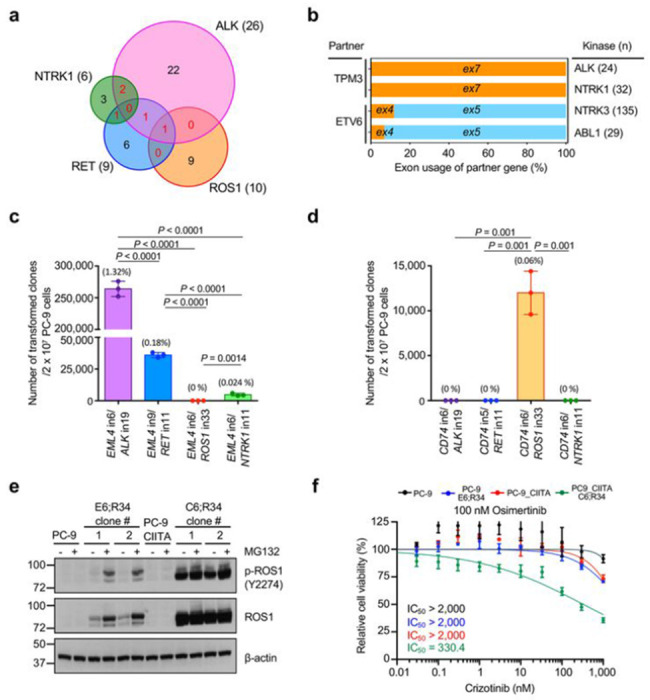
The fusion partners of different TKs are largely exclusive. **a**, Venn diagrams showing the overlap of fusion partners shared between *ALK* (n=26 partner genes), *RET* (n=9), *ROS1* (n=10), and *NTRK1* (n=6) fusions identified by FACTS. **b**, Comparisons of exon usages of partner genes between different kinase genes. **c**, Quantification of osimertinib-resistant clones in PC-9 cells with fusions induced by between EML4 and different TKs. Data show means of three biological replicates, with error bars representing ±s.e.m; significance was determined by an unpaired, two-tailed Student’s *t*-test. **d**, Quantification of osimertinib-resistant clones in PC-9 cells with fusions induced by between CD74 and different TKs. Data show means of three biological replicates, with error bars representing ±s.e.m; significance was determined by an unpaired, two-tailed Student’s *t*-test. **e**, Representative western blot analysis of EML4-ROS1 and CD74-ROS1 fusions after MG-132 treatment. Similar results were observed in two independent experiments. **f**, Sensitivity to combination of osimertinib plus crizotinib in clones with EML4-ROS1 E6;R34 and CD74-ROS1 C6;R34 fusions. Data show means of three biological replicates, with error bars representing ±s.e.m.

Next, we investigated the process of TK fusion selection. Consistent with the COSMIC analysis in patients, TK fusion partners obtained by FACTS were mutually exclusive in most cases^30,40–42^, with only a few partners shared by multiple TK fusions (Fig. 4a **and Supplementary Table 4**). Interestingly, some fusion partner genes, such as *TPM3* and *ETV6*, used the same exons when they generated oncogenic fusions with different TKs (Fig. 4b). Thus, we explored functional basis of fusion-partner specificity to each kinase. We engineered all combinations of EML4 and CD74 fusions with ALK, RET, ROS1, and NTRK1 (**Extended Data Fig. 8a, b**). While all of the fusion junctions were detected equally at the genomic DNA levels (**Extended Data Fig. 8c,d**), some of the kinase fusion combinations did not yield resistant clones under osimertinib selection (Fig. 4c,d). While thousands of EML4-ALK, EML4-RET, or EML4-NTRK1 clones rapidly emerged, no clones with EML4-ROS1 fusions were observed (Fig. 4c). Likewise, while thousands of CD74-ROS1 clones emerged, no clones with CD74-ALK, CD74-RET, or CD74-NTRK1 fusions emerged in PC9 cells expressing CIITA (Fig. 4d). Next, we isolated single cell-derived clones harboring different fusions for further characterization (**Extended Data Fig. 8e**). Clones with CD74-ROS1 fusion displayed abundant protein that was phosphorylated as expected, but clones with EML4-ROS1 fusion showed very low abundance of the EML4-ROS1 protein that was also poorly phosphorylated (Fig. 4e). Treatment with proteasome inhibitor MG132 stabilized the EML4-ROS1 fusion protein and its phosphorylation substantially increased (Fig. 4e). Crizotinib, primarily a MET inhibitor with an activity on ROS1, inhibited the growth of clones harboring CD74-ROS1 fusions but not EML4-ROS1 fusions, suggesting that only stable and abundant kinase fusions could create oncogenic dependency (Fig. 4f).

### Protein stability determines the specific exon usage of oncogenic TK fusions

**Figure 5:**
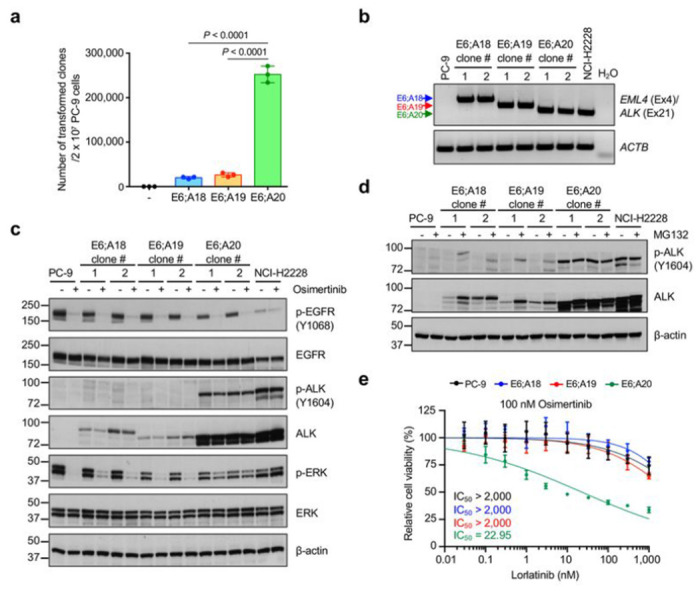
EML4-ALK fusions have the preferential usage of selected exons. **a,** Quantification of osimertinib-resistant clones in PC-9 cells carrying different EML4-ALK fusion variants. Data show means of three biological replicates, with error bars representing ±s.e.m; significance was determined by an unpaired, two-tailed Student’s *t*-test. **b**, mRNA expression of EML4-ALK transcripts by RT-PCR. **c**, Representative western blots of the signaling changes in different EML4-ALK fusion variants treated with EGFR inhibitor (osimertinib). Similar results were observed in two independent experiments. **d**, Representative western blot analysis of EML4-ALK fusion variants after MG-132 treatment. Similar results were observed in two independent experiments. **e**, Sensitivity to combination of osimertinib plus lorlatinib in clones with different EML4-ALK fusion variants. Data show means of three biological replicates, with error bars representing ±s.e.m.

An additional finding of COSMIC analysis was the preferential usage of specific exons in TK fusions (Fig. 1e, f). To better understand molecular basis of preferential exon usage in kinase fusions, we engineered EML4-ALK variants by CRISPR/Cas9 that fuse the same *EML4* exon 6 to different *ALK* exons (e18, e19, or e20) (**Extended Data Fig. 8f**). All these fusions are predicted to be in frame, which could potentially lead to functional ALK fusions. However, these different fusion variants have been detected in different frequencies in patients, the EML4-ALK E6;A20 fusion being far more frequent than the E6;A18 or E6;A19 fusions (less than 1% among ALK fusions)^46,47^ (Fig. 1e), which was consistently observed in FACTS (Fig. 5a). To understand the cause of these differences, we generated clonal lines for each fusion variant. The mRNA transcription levels were comparable among the variants, likely due to their regulation by the same promoter (Fig. 5b **and Extended Data Fig. 8g**). However, the protein abundance and the level of phosphorylation were markedly different (Fig. 5c **and Extended Data Fig. 8h**). The E6;A20 fusion protein was highly expressed and phosphorylated, whereas the E6;A18 or E6;A19 fusions were much less abundant with barely detectable phosphorylation (Fig. 5c and **Extended Data Fig. 8h**). Consequently, the E6;A20 variant showed a greater potency in rescuing MAPK pathway activation compared to the E6;A18 or E6;A19 fusions in osimertinib-treated PC-9 cells (Fig. 5c). Treatment with MG132 stabilized the E6;A18 or E6;A19 fusions and led to their phosphorylation (Fig. 5d). Next, we investigated whether the different functional features of these EML4-ALK fusion variants were due to differences in subcellular localization, given recent evidence showing that the oncogenic activity of EML4-ALK is dependent on its subcellular localization and formation of protein granules in the cell cytoplasm^48^. The three EML4-ALK fusion variants showed comparable intracellular localization in confocal microscopy analysis (**Extended Data Fig. 8i, j**), with weaker signals with the E6:A18 or E6:A19 fusions, likely due to their low protein abundance. Functional assay showed that lorlatinib inhibited the growth of cells harboring E6;A20 fusions but not of the E6;A18 and E6;A19 fusions, suggesting that only E6;A20 fusions are stable enough to confer oncogenic dependence (Fig. 5e). These findings imply that the usage of specific exons in TK fusions is likely dictated by protein stability rather than transcription or subcellular localization of the resulting fusions, and that only an abundant expression of TK fusion proteins creates a dependency that might determine the efficacy of TKI treatment.

### TKI therapy is less effective in patients with atypical ALK fusions

**Figure 6:**
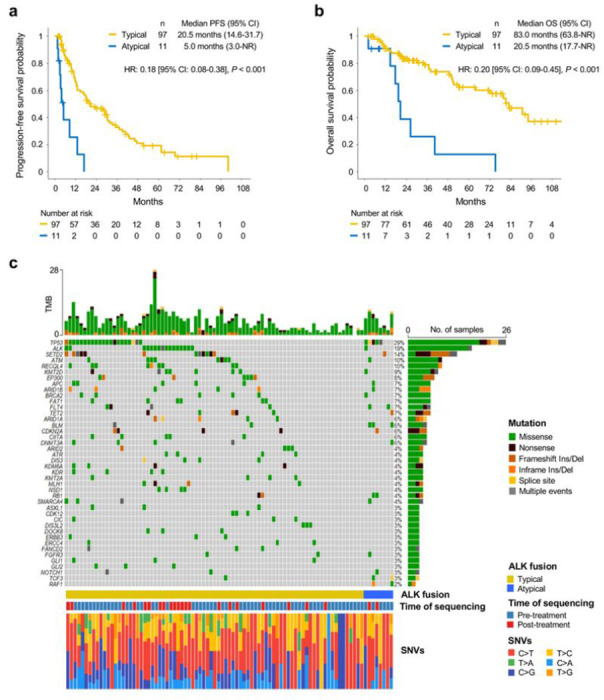
Atypical ALK fusions show a poor survival compared to typical ALK fusions. **a and b,** Progression-free (**a**) and overall (**b**) survival in patients carrying typical and atypical ALK fusions treated with ALK inhibitors. **c**, OncoPrint plot showing the top 42 genes mutated among NSCLC harboring typical and atypical ALK fusions.

Since atypical ALK fusions showed reduced functionality and oncogenic signaling in PC-9 cell models (Fig. 5c, e), we investigated whether these findings were reflected in patients by studying clinical responses to ALK TKIs in patients carrying either typical or atypical ALK fusions. We analyzed 108 patients with metastatic NSCLC who tested positive for ALK fusions by next-generation sequencing (NGS) and received ALK TKI treatment and divided them into two groups based on the *ALK* gene fusion breakpoints: typical (breakpoints in *ALK* intron 19) and atypical (breakpoints in other *ALK* introns/exons or atypical fusion partner). There were 97 typical ALK fusions with *ALK* breakpoints in intron 19, and 11 atypical fusions cases with breakpoints in introns 16, 17, 18, and 20 or inside exon 20 (**Extended Data Fig. 9a and Supplementary Table 8**). Patients with atypical ALK fusions had clinical characteristics comparable to patients with typical ALK fusions in terms of age, gender, smoking history, ECOG performance status, and ALK inhibitor treatment (**Extended Data Fig. 9b**). The typical ALK fusion group had 88.7% (88/97) of EML4-ALK fusions or other known oncogenic ALK fusions, such as HIP1-ALK^49^, whereas the group of atypical ALK fusions was composed of 54.5% (6/11) of EML4-ALK fusions with non-intron 19 breakpoints (**Extended Data Fig. 9c**) or ALK fusions with atypical partners. Strikingly, the atypical group showed significantly lower objective response rate (ORR) to ALK TKI compared to the group of patients with typical ALK fusions (54.5% versus 88.7%, *p* = 0.01) (**Extended Data Fig. 9d**), resulting in a significantly shorter progression-free survival (PFS; 5 months versus 20.5 months, HR: 0.18 [95% CI: 0.08–0.38], *p* < 0.001) and overall survival (OS; 20.5 months versus 83.0 months, HR: 0.20 [95%CI: 0.09–0.45], *p* < 0.001) (Fig. 6a,b). We also confirmed that atypical ALK fusions retained a significant association with shorter PFS and OS after adjusting for potential confounders in multivariable Cox regression models (**Extended Data Fig. 9e**). We further examined co-occurring mutations in cases with typical or atypical ALK fusions. The most frequently mutated gene was *TP53* in typical and atypical ALK fusions, with a significantly higher frequency in atypical fusions (62.5% versus 25.9%, *p* = 0.046), which may have also contributed to the worse outcomes to ALK TKIs observed in this subset of patients (Fig. 6c). In addition, atypical ALK fusions were associated with a higher rate of mutations in alternative oncogenic driver genes, including *BLM, FLT4, RAFT, RB1*, and *TCF3*^51^, compared to typical ALK fusions (Fig. 6c **and Extended Data Fig. 9f**). Overall, these results demonstrate that atypical TK fusions are weaker oncogenic driver, are associated with increased co-mutation of other oncogenes, and respond poorly to ALK inhibition, providing a biomarker predictor for response to ALK TKI in patients.

## Discussion

In this work we provide mechanistic explanation and clinical relevance of the recurrent patterns of oncogenic TK fusion in cancers. TK fusions show mutually exclusive fusion partners, with just a few partner genes shared by multiple TK genes. In addition, TK and partner genes employ a preferential usage of specific exons.

To explain these patterns, we developed FACTS as a novel approach to identify genome-wide functional chromosomal translocations that drive solid tumor growth and resistance to TKI inhibition. Current techniques to map genome-wide chromosomal translocations are mostly focused on early, unbiased mechanistic events of DNA translocation formation largely in B lymphocytes^17,18^, without interrogating the oncogenicity of the cloned translocations. In contrast, by FACTS we found that oncogenic ALK, RET, ROS1, and NTRK1 fusions form spontaneously in normal or tumoral lung epithelial cells when a DSB is introduced in the TK gene. These spontaneous translocations occur not only in PC-9 lung cancer cells selected *in vitro* by the pressure of osimertinib, but also in non-tumoral BEAS-2B cells that are transformed *in vivo* in mice, indicating that FACTS can be applied to tumoral or non-tumoral cells subjected to different selection modalities. By extension, it is conceivable that FACTS could be applied virtually to almost any TK gene or any normal or tumoral cells for which a method of selective pressure is available. Furthermore, it is likely that FACTS application could be expanded to non-TK fusions, such as translocations recurrently found in sarcomas, hematologic malignancies, or other tumors.

By applying FACTS to lung epithelial cells, we discovered key factors leading to the formation of chromosomal translocations in solid tumors. We found that mRNA expression level of the partner gene was essential to the point that reactivation of transcription, such as in the case of *HLA-DRB1*, was sufficient to induce the spontaneous formation of functional fusions (**Extended Data** Figs. 4 and 5). Gene transcription was required not only to express the resulting fusion but also to increase the probability of DSBs occurring within a gene, in keeping with the knowledge that transcription levels in a gene correlate with DSB frequency^52,53^. This mechanism has implications for the understanding of the cell of origin in lung cancers driven by chromosomal translocations. For example, lung cancers with HLA-DR or CD74 fusions are most likely to originate in cells that express these genes robustly, such as alveolar type II cells^54,55^ (**Extended Data Fig. 10a-c**).

By comparing patterns of rearrangements before selection by HTGTS to those after selection by FACTS, we further gained insights in the process of oncogenic TK fusion formation. Before selection, HTGTS detected no clusters of DSB breakpoints in TK genes (**Extended Data Fig. 7b-j**) and only few clusters in partner genes (Fig. 3k **and Extended Data** Fig. 6e-g), suggesting that most TK fusions originate by a selection of translocations that arise from spontaneous DSBs dispersed throughout the genome without pre-determined hotspots. These DSBs are likely generated by various mechanisms, including the formation of R-loops, G4 quadruplex, stalled replication forks, correlate with active transcription^56^ and might be facilitated by enzymatic activity of APOBEC enzymes^57^. Thus, in solid tumors recurrent translocations might be selected by different mechanisms than in hematologic malignancies, such as B-cell lymphoma, in which recurrent translocation are largely dictated by the off-target activity of the activation-induced cytidine deaminase (AID) and the recombination activating gene (RAG)1/2 enzymes^52,58,59^. Furthermore, recurrent TK translocations do not appear to occur at a high frequency when compared to other translocations, but oncogenic translocations are heavily selected due to their potential to drive cancer cell survival and proliferation.

During the selection process, only genomic breakpoints located in introns with the correct orientation that result in fusion proteins with functional activity were enriched, whereas breakpoints leading to out-of-frame proteins disappeared. Proximity of the genes also played a role because we consistently identified fusions with genes located on the same chromosome that constitute a topological domain with a higher probability of contact^44,45,60^. While the generation of in-frame and highly transcribed fusions with a partner that have dimerization domains are expected mechanistic factors, they are insufficient to explain the specific exon usage or partner usage of each TK fusion as it is observed in patients. For example, EML4 is the most frequent fusion partner for ALK, but it is very rarely translocated with RET, ROS1 or NTRK1; likewise, CCDC6-RET and CD74-ROS1 fusions are frequently found in NSCLC patients, but CCDC6 fusion with ALK or ROS1 and CD74 fusion with ALK have never been described^27,35–37^ (**Extended Data Fig. 10d-g**). We discovered that protein stability is a key determinant factor that could be not predicted simply based on the characteristic of the fusion partner. By investigating EML4-ALK fusions with different *ALK* exons, we demonstrated that a specific exon usage was critical to provide stability to the fusion protein (Fig. 5). We found that inhibition of the proteasome by MG-132 in the unstable EML4-ALK E6;A18 and E6;A19 variants increased protein abundance (Fig. 5d). Recent study suggested that degrons, short motifs that affect protein degradation rate (i.e. D box, KEN box, SPOP motif, and PEST sequences), could regulate the expression of fusion proteins by the degron loss mechanism^61^. PEST sequences^62^ are present in the unstable EML4-ALK E6;A18 and E6;A19 variants but not in the stable oncogenic EML4-ALK E6;A20 variant, implying that degron gain in the EML4-ALK E6;A18 and E6;A19 variants might contribute to their rapid degradation through the ubiquitin-proteasome pathway. Similarly, we observed that oncogenic fusions were stable only when each TK portion was paired with a specific partner but with other partners (Fig. 4). While the CD74-ROS1 fusion was stable and phosphorylated, the EML4-ROS1 fusion, which is not seen in patients, was unstable and poorly phosphorylated (Fig. 4e). Thus, the selection process of the specific fusion partner for each TK depends not only on the availability of an in-frame dimerization domain but also on the stability of the resulting fusion. Furthermore, the pattern of recurrent oncogenic TK fusions may be dictated by the protein stability of the resulting fusion proteins rather than the enriched DSBs in specific locations in the cancer genome.

Accurate detection of oncogenic TK fusions is critical to effective treatment for cancer patients with TK fusions. Several methods, such as fluorescence in situ hybridization (FISH), immunohistochemistry (IHC), quantitative real-time PCR (qRT-PCR), targeted DNA sequencing, and targeted RNA sequencing, are routinely employed to diagnose TK fusions. Although these techniques are sufficient for detecting TK fusions, they do not provide functional evidence for their oncogenic activity. Indeed, recent studies showed that some patients do not respond to ALK TKIs despite IHC and NGS confirmation of ALK fusions^63,64^. We demonstrate here that the stability and functionality of the TK fusion proteins is a key factor for the TKI response (Figs. 4 and 5), indicating that the detection of TK fusion junctions by DNA- and RNA-based sequencing does not fully predict the response to TKI therapy. We showed that patients with atypical EML4-ALK rearrangements encoding for unstable fusion proteins responded poorly to ALK TKIs and had shorter PFS and OS compared to patients with typical EML4-ALK (Fig. 6a, b **and Extended Data Fig. 9d**). Although these data are still limited by the small sample size, they suggest that a strong oncogenic dependency by tumor cells develops only when the TK fusion is stably expressed, and that this dependency predicts TKI response in patients. Thus, studies on novel ALK inhibitors should stratify results by TK junction as patients with atypical ALK junctions may not respond as well.
